# Genome-Wide Identification of the *ClpB* Gene Family in Tomato and Expression Analysis Under Heat Stress

**DOI:** 10.3390/ijms252212325

**Published:** 2024-11-17

**Authors:** Yuemei Zhang, Tailai Yang, Jiaxi Han, Xiao Su, Yanqing Cong, Ming Zhou, Yan Wang, Tao Lin

**Affiliations:** 1 Department of Vegetable Science, College of Horticulture, China Agricultural University, Beijing 100193, China; zym941128@163.com (Y.Z.); 18813037316@163.com (T.Y.); h_tevanm@163.com (J.H.); suxiao0211@163.com (X.S.); cyq913245036@163.com (Y.C.); 2 Key Laboratory of Biology and Genetic Improvement of Horticultural Crops (North China), Ministry of Agriculture, Beijing Vegetable Research Center, Beijing Academy of Agriculture and Forestry Sciences, Beijing 100097, China; zhouming1981@126.com

**Keywords:** Caseinolytic Protease B, *Solanum lycopersicum*, high temperature, expression pattern, AAA+

## Abstract

Tomato is a widely grown horticultural crop, and its growth process is often affected by high temperatures. Caseinolytic Protease B (ClpB), a homologous protein to heat shock protein 101 (HSP101), plays a vital role in plant heat adaptation and development. In this study, we identified six *SlClpB* genes in tomatoes, distributed across four chromosomes. Collinearity analysis revealed that the gene pairs *SlClpB-2* and *SlClpB-3A*, as well as *SlClpB-3C* and *SlClpB-12*, resulted from segmental duplication events. Phylogenetic and motif analyses showed that ClpB proteins possess highly conserved domains across different species. We used RNA-seq data to analyze the expression patterns of the ClpB family. Among them, *SlClpB-3A* and *SlClpB-12* exhibited increased expression in multiple tissues under heat stress. Specifically, *SlClpB-2*, *SlClpB-3A*, and *SlClpB-3C* were highly expressed in the fruit orange stage and in flower buds under heat treatment, while in seedlings, *SlClpB-2* and *SlClpB-3A* exhibited heat-induced expression. Real-time quantitative fluorescent PCR (qRT-PCR) results showed that the expression of *SlClpB-2* and *SlClpB-3A* was significantly increased under heat stress in the leaves and buds of Ailsa Craig, Micro-Tom, and M82. Overall, our findings provide valuable insights into the regulatory mechanisms of *SlClpB* genes in response to heat stress.

## 1. Introduction

Caseinolytic Protease B (ClpB) is a member of the AAA+ protein family and functions as a protein disaggregase; it is crucial for refolding denatured proteins during heat stress. By preventing protein aggregation and irreversible denaturation, ClpB plays an essential role in maintaining protein functionality and enhancing cellular survival under heat stress [1,2,3,4,5]. Structurally, ClpB features a spherical N-terminal domain, two AAA+ modules, and an extended coiled-coil M-domain [6]. The N-terminal domain, while flexible and involved in substrate interactions, is not essential for disaggregation activity [7,8,9]. In contrast, the AAA+ modules are indispensable for ATP binding and hydrolysis, forming the core of the hexameric ring structure. During ATP hydrolysis, these modules undergo significant conformational changes, transitioning from a ring-shaped structure to helices or twisted half helices. This structural transformation facilitates ClpB proteins to form oligomers, which is key to ClpB’s ability to resolve protein aggregates [10,11,12,13].

The ClpB protein’s primary structure can be divided into five regions: nucleotide-binding domains (NBDI and NBDII), the N-terminal, the C-terminal, the Sensor and Substrate Discrimination (SSD) domain, and the middle region [5]. NBDI and NBDII are conserved functional regions, each containing a core nucleotide-binding site with Walker A and Walker B motifs. Walker A is essential for nucleotide binding and drives chaperone activity, whereas the Walker B motif facilitates ATPase activity by interacting with casein [11]. The middle region forms an elongated coiled-coil structure that links NBDI and NBDII and plays a crucial role in the chaperone function [14]. The N-terminal and C-terminal regions are primarily involved in supporting ClpB oligomerization [15,16]. The SSD domain is responsible for substrate recognition by energy-dependent proteases [17].

ClpB proteins are vital in various biological processes, particularly in stress responses and developmental stages. As members of the heat shock protein (HSPs) family, their expression is upregulated in response to elevated temperature [18,19,20]. ClpB protein is the most important conserved protein in the development of heat-resistant stress in plants [21]. In tobacco plants, the combination of drought and heat stress upregulated *ClpB* expression [22]. Studies on HS104, a yeast homolog of ClpB, have highlighted its crucial function in heat adaptation and stress resistance [1,23,24]. In plants, the ClpB homolog HSP101 shares considerable similarity with yeast HSP104 and is predominantly localized in the cytoplasm. This protein has been identified in several plant species, including *Arabidopsis thaliana*, soybean (*Glycine max*), maize (*Zea mays*), wheat (*Triticum aestivum*), and tobacco (*Nicotiana tabacum*) [25,26,27,28]. HSP101 is especially noted for its role in plastid development and stress tolerance [29]. In lima beans, the expression of *HSP101*, also known as PlHSP100, increases at 37 °C [30]. Additionally, the Arabidopsis mutant *hot1-1*, which carries a mutation in the *ClpB1/Hsp101* allele, exhibits decreased heat resistance in seedlings and a diminished ability to survive high temperatures in germinated seeds [26]. HSP101 also facilitates the accumulation of *HSA32* after heat acclimation, slowing the degradation of HSP101 and thereby aiding in the development of acquired thermotolerance, which is crucial for plant adaptation to temperature fluctuations [31]. OsHSP101 forms a stable complex at high temperatures and plays an important role in improving the heat resistance of rice [32]. Molecular evolutionary analysis indicates a high degree of conservation in the cytoplasmic sequences of *HSP101* across species [26,28].

Tomato (*Solanum lycopersicum* L.) is a major horticultural crop and a valuable plant research model organism encompassing approximately 35,000 genes in its genome [33,34,35]. As sessile organisms, plants are constantly exposed to changes in temperature [36]. Plants have developed complex and diverse defenses against heat stress [37]. ClpB homologs in tomatoes play a key role in heat adaptation and stress responses [38]. As immobile organisms fixed to the soil, plants, including tomatoes, must adapt to changing environmental conditions. These adaptations are achieved through mechanisms such as phenotypic plasticity, which enables a single genotype to display different phenotypes in various environments [39]. Understanding the functions of different gene families, particularly those associated with stress responses, is essential for enhancing our understanding of plant growth, development, and adaptability.

This study aims to conduct a comprehensive genome-wide analysis of the ClpB family in tomatoes, with a focus on their phylogenetic relationships and evolutionary history. We identified six potential *SlClpB* genes in tomatoes, paving the way for further exploration into the functional diversity and heat response mechanism of the ClpB family in this species.

## 2. Results

### 2.1. Identification of SlClpB Genes

In Arabidopsis, ClpB proteins are well characterized, including cytoplasm-targeted ClpB (*AtClpB1*), chloroplast-targeted ClpB (*AtClpB3*), and mitochondria-targeted ClpB (*AtClpB4*). *AtClpB2*, another chloroplast-targeted gene, lacks a specific ClpB protein structure (ClpB-D2) and was therefore excluded from our analysis. Using these three Arabidopsis ClpB proteins as reference sequences, we identified six *ClpB* genes in the tomato genome (SL4.0 build), which were renamed according to their chromosome locations in this study (Table 1). For instance, the Solyc02g088610 gene, previously known as *SlLeHSP100* [40], is here referred to as *SlClpB-2*. The molecular weights of these SlClpB proteins range from 95.071 to 110.377 kDa, with protein lengths varying between 854 and 980 amino acids (aa). Their isoelectric points (pI) span from 4.82 to 6.88. Signaling peptide prediction indicated the absence of signaling peptides in these proteins, suggesting that their primary functions may occur within the cytoplasm or nucleus. Subcellular localization prediction further revealed that *SlClpB-2* was located in the chloroplast, while the others were localized in the cytoplasm (Table 1).

### 2.2. Systematic Profiles of SlClpB Genes

**Figure 1:**
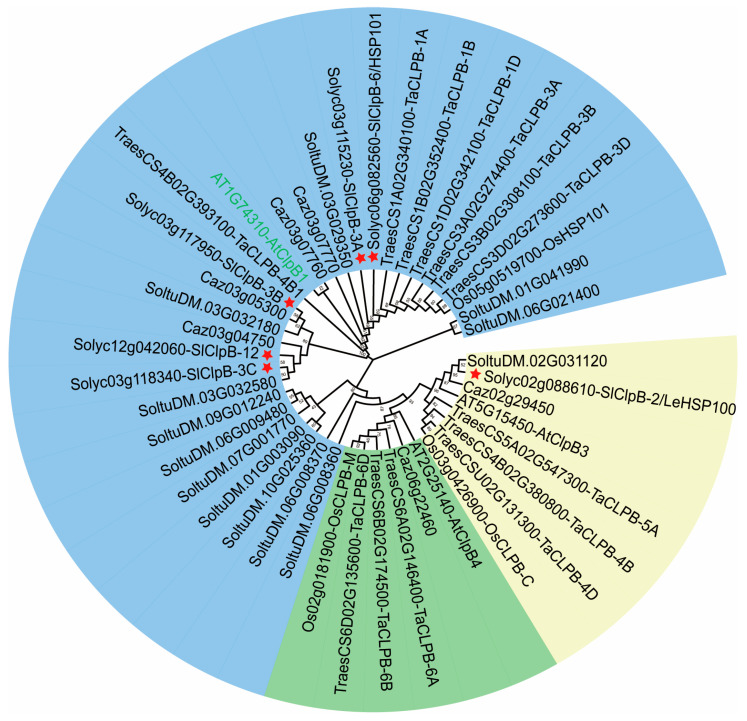
Phylogenetic analysis of the ClpB family. This figure illustrates the phylogenetic relationships among ClpB proteins from various species, including *Arabidopsis thaliana* (*At*), *Capsicum annuum* (*Ca*), *Solanum lycopersicum* (*Sl*), *Solanum tuberosum* (*St*), *Triticum aestivum* (*Ta*), and *Oryza sativa* (*Os*). Bootstrap values from 1000 replicates are shown at the nodes. Asterisks indicate SlClpB family members.

To explore the relationships of ClpB proteins across species, we analyzed protein sequences from tomato (*Solanum lycopersicum*), pepper (*Capsicum annuum*), and potato (*Solanum tuberosum*), along with members from Arabidopsis (*Arabidopsis thaliana*), rice (*Oryza sativa*), and wheat (*Triticum aestivum*) (Table S1). Phylogenetic analysis using the maximum likelihood method revealed that the ClpB family comprises three different clades. Five *SlClpB* genes were grouped with *AtClpB1*, which is located in the cytoplasm in Arabidopsis, while one *SlClpB* gene, *SlClpB-2*, clustered with the chloroplast *AtClpB3* (Figure 1). These results were consistent with the predicted subcellular localizations. No mitochondrial homologs were identified. This analysis provides insight into the evolutionary relationships among ClpBs in plant species, highlighting their diversification and adaption to various subcellular compartments.

### 2.3. Evolutionary Investigation of SlClpB Genes

**Figure 2:**
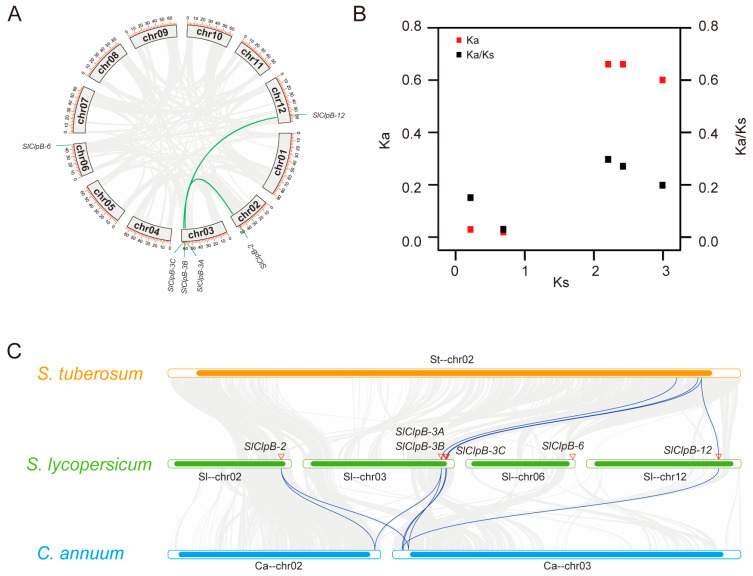
Genome localization and collinearity analysis of *SlClpBs:* (**A**) Synteny analysis of *SlClpBs* in *Solanum lycopersicum* genome. The number and length of chromosomes are displayed. The green lines represent gene synteny relationships. (**B**) Ka, Ks, and Ka/Ks ratio calculation of *SlClpBs*. (**C**) Genome-wide synteny analysis of *SlClpBs* in different species genomes. Synteny analysis between *Solanum tuberosum* (*S. tuberosum*), *Solanum lycopersicum* (*S. lycopersicum*), and *Capsicum annuum* (*C. annuum*). Blue lines represent the orthologous genes of SlClpB genes.

To investigate the evolutionary relationships within the ClpB family, we analyzed various evolutionary processes in the tomato genome (Figure 2). Our synteny analysis identified two gene pairs—*SlClpB-2* with *SlClpB-3A*, and *SlClpB-3C* with *SlClpB-12*—that likely originated from a single ancient whole-genome duplication event (Figure 2A). To further explore the evolutionary relationships of the six *SlClpB* genes, we performed a Ka/Ks analysis on their nucleic acid sequences, finding Ka/Ks values ranging from 0.03 to 0.3 (Table S2; Figure 2B). These results suggest that the SlClpB family’s evolution is likely influenced by purifying selection, which eliminates deleterious nonsynonymous mutations to maintain gene function. For a broader perspective, we performed collinearity analysis with *ClpB* genes from pepper and potato plants. We identified 13 collinear gene pairs: seven between peppers and tomatoes and six between potatoes and tomatoes (Figure 2C). Notably, *SlClpB-6* was unique to *Solanum lycopersicum*, with no collinear counterparts in *Solanum tuberosum* and *Capsicum annum*.

### 2.4. Conserved Motifs and Domains of SlClpB Genes

**Figure 3:**
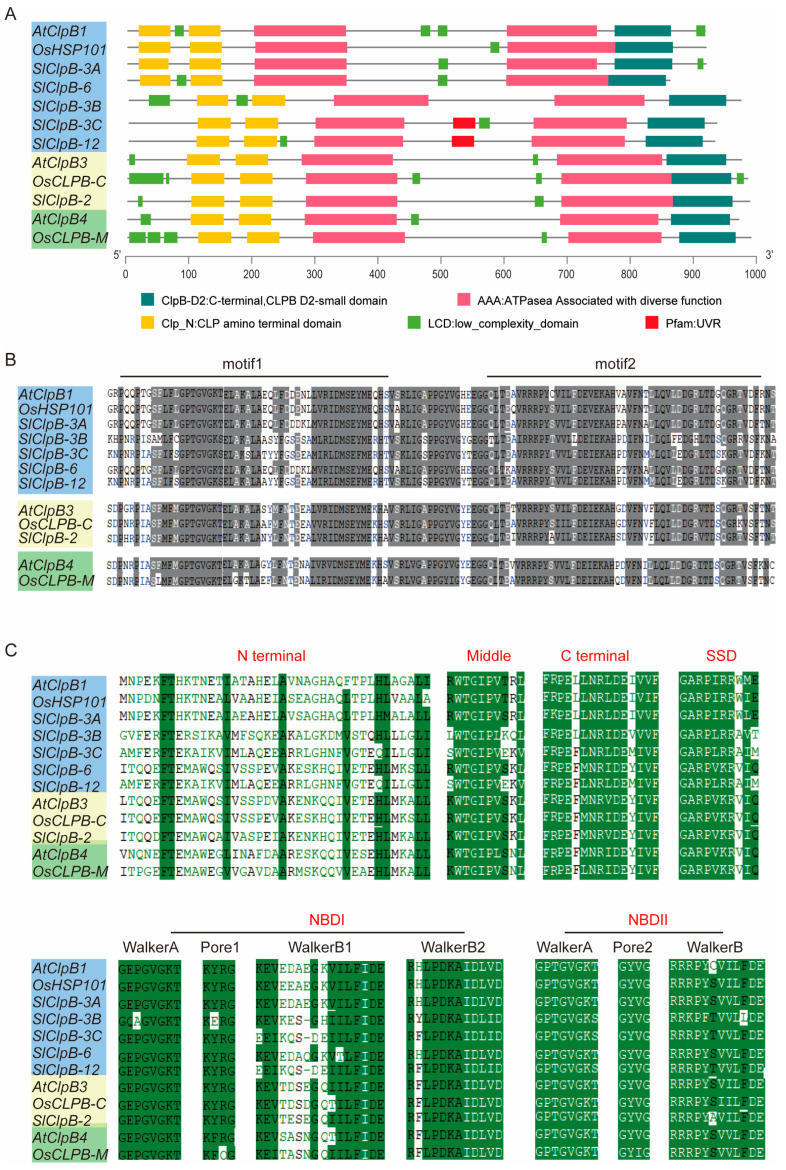
Conserved domains and motifs identified in ClpB proteins. (**A**) Illustration of the conserved domains present in ClpB proteins from Arabidopsis, rice, and tomato. (**B**) Variability in the residues of the middle motifs among ClpB proteins from species in (**A**). Conserved residues are highlighted in white with a grey background, semi-conserved residues in black with a grey background, and non-conserved residues are in different colors without shading. (**C**) Unique sequence features of ClpB proteins from Arabidopsis, rice, and tomato. Consensus sequences highlight key residues in both cytoplasmic and plastid-targeted ClpBs across conserved regions, including the N-terminal, middle, and C-terminal domains, the SSD (Sensor and Substrate Discrimination) motif, and NBDs (nucleotide-binding domains) I and II. Residues shared among the three species are marked with a grey background. Conservative residues are depicted in white with a green background, semi-conservative residues are in black with a green background, and non-conserved residues are in different colors without shading. The blue, yellow, and green background colors of SlClpB genes are consistent with the phylogenetic relationships in SlClpB family members.

To assess the conservation of SlClpB family proteins, we visualized their gene structures and identified conserved motifs and domains within the protein sequences, with *Arabidopsis thaliana* serving as the reference. The results showed three distinct domain clusters in all SlClpB proteins: (1) two ATPase associated with diverse function domains (AAA); (2) two Clp amino-terminal domains (Clp_N); and (3) one C-terminal D2-small domain (Clp_D2-Small) (Figure 3A).

In ClpB proteins, the middle region forms a coiled-coil structure with several identified domains (Figure 3A), such as motifs associated with UVR (Ultraviolet B photoreceptor) and/or low-complexity domains (LCD). Our analysis of ClpB motifs in Arabidopsis, rice, and tomato revealed two conserved motifs, namely motif 1 and motif 2 (Figure 3B). We observed high sequence conservation among ClpB proteins within the same subcellular compartments, except SlClpB-3B, which displayed lower similarity to the other chloroplast ClpBs. At the sequence level, specific residues that differentiate ClpB from other ATPase family members were identified in all SlClpB proteins with minor variations (Figure 3C). These key residues and motifs include the N-terminal region; NBDI, Walker A, Walker B1, Walker B2; the middle domain; NBDII, Walker A, Walker B; and the C-terminal end. These residues and motifs are important for ClpB functionality, as they affect ATPase activity and substrate binding across diverse plant species.

**Figure 4:**
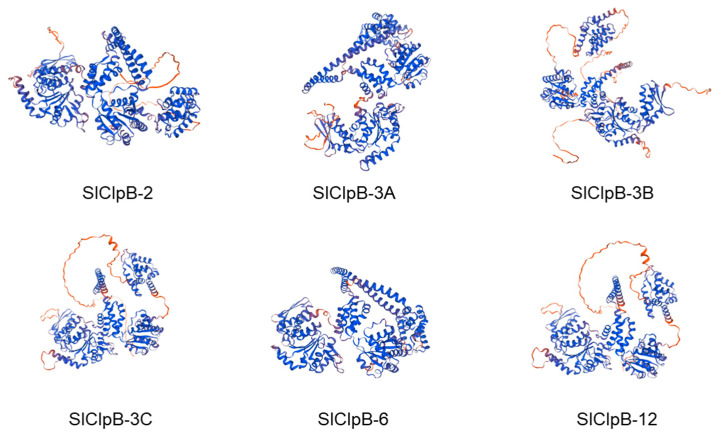
The 3D structure of SlClpB proteins. Blue indicates high confidence in the forecast results, while red indicates low confidence in the forecast results.

In order to further understand the protein structure, we predicted the 3D structure of the tomato ClpB proteins. We found that the protein structure was mainly composed of random coils and α-helical and β-folded structures (Figure 4). This indicates that tomato’s ClpB protein structure is certainly conserved.

### 2.5. Identification of Cis-Acting Elements of SlClpB Promoters

**Figure 5:**
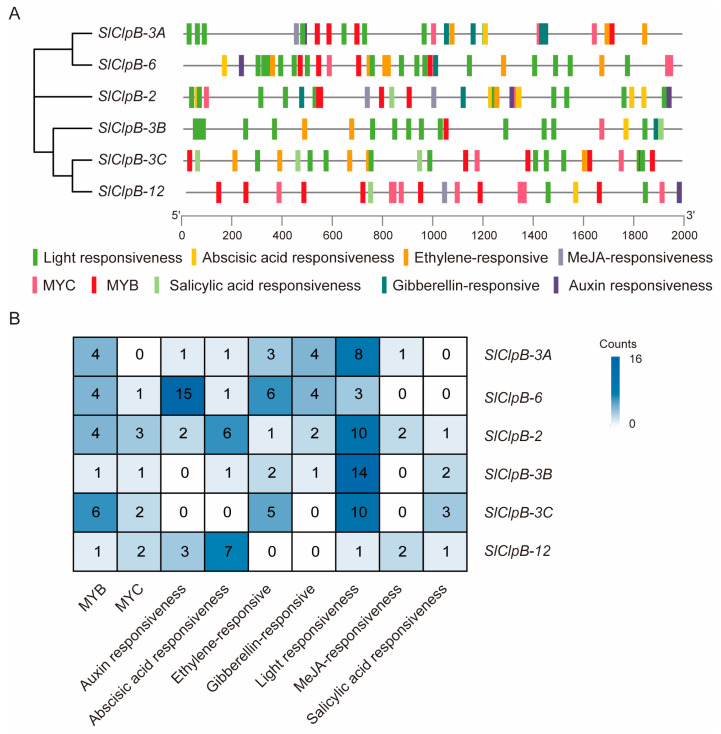
Cis-acting elements in the promoters of *SlClpB* genes. (**A**) Identification of promoter regulatory cis-acting elements in *SlClpB* genes. (**B**) The quantities and types of cis-acting elements present in the *SlClpB* promoters.

Many studies have emphasized the significant role of the ClpB family in plants. Understanding the regulatory cis elements in their promoters can shed light on gene function. To investigate the mechanisms that regulate the expression of *SlClpB* genes, we analyzed the promoter sequences of the *SlClpB* family genes (Figure 5). Our analysis revealed that the majority of *SlClpB* promoters contain light-responsive elements, transcription factor binding sites, and hormone-responsive elements.

Our analysis revealed that all six *SlClpB* genes had light responsiveness and MYB elements on their promoters. *SlClpB-2* and *SlClpB-6* both contain auxin, abscisic acid, gibberriceellin, and methyl jasmonate response elements. All *SlClpB* promoters except *SlClpB-3C* contain abscisic acid response elements (Figure 5A). Light-responsive elements are the most common, with a total of 46 identified across the ClpB family, and are particularly abundant in *SlClpB-3B*, which has 14 such elements (Figure 5B). Hormone-responsive elements are also numerous, including those responsive to jasmonic acid methyl ester, abscisic acid, gibberellin, salicylic acid, and ethylene. Notably, *SlClpB-6* has the highest number of auxin-responsive elements, totaling 15 (Figure 5B). These findings suggest that the SlClpB family may respond to a range of hormonal signals, indicating the complexity of their regulatory networks (Figure 5).

### 2.6. Expression Pattern of SlClpB Genes

**Figure 6:**
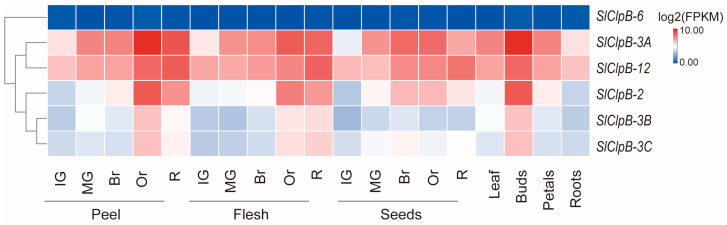
Expression patterns of the *SlClpB* gene family in Micro-Tom. RNA-seq data were analyzed using the Tom Express browser to examine the expression of *SlClpB* genes across different developmental stages in various tissues of tomato. The data reveal expression levels in root, leaf, flower bud, flower petal, and fruit. Br, breaker; IG, immature green; MG, mature green; Or, orange; R, ripening. FPKM, fragments per kilobase of transcript per million mapped reads.

To assess the role of *SlClpB* genes in tomato development, we performed a gene expression analysis using RNA-seq data obtained from the cultivar Micro-Tom. This analysis included samples from seeds, roots, leaves, flower buds, petals, and fruits [41]. Gene expression levels were log-transformed and normalized to generate a heatmap (Figure 6). The results showed that in fruit peels, *SlClpB-2*, *SlClpB-3A*, and *SlClpB-12* were significantly upregulated during both the orange and ripening stages. However, *SlClpB-3B* and *SlClpB-3C* were upregulated at the orange stage after being downregulated at the ripening stage. In fruit flesh, *SlClpB-2*, *SlClpB-3A*, *SlClpB-3B*, *SlClpB-3C*, and *SlClpB-12* were upregulated during both the orange and ripening stages. During the development of seeds, *SlClpB-2* and *SlClpB-3A* showed increased expression at the mature green, breaker, and orange stages, whereas *SlClpB-12* expression rose at the breaker, orange, and ripening stages (Figure 6). Notably, *SlClpB-3A* exhibited the highest level of upregulation, indicating its significant role in fruit development across various stages. In leaves, buds, petals, and roots, all genes except *SlClpB-6* exhibited significant upregulation in flower buds. These findings suggest that the SlClpB family is likely involved in the development of both flowers and fruits.

**Figure 7:**
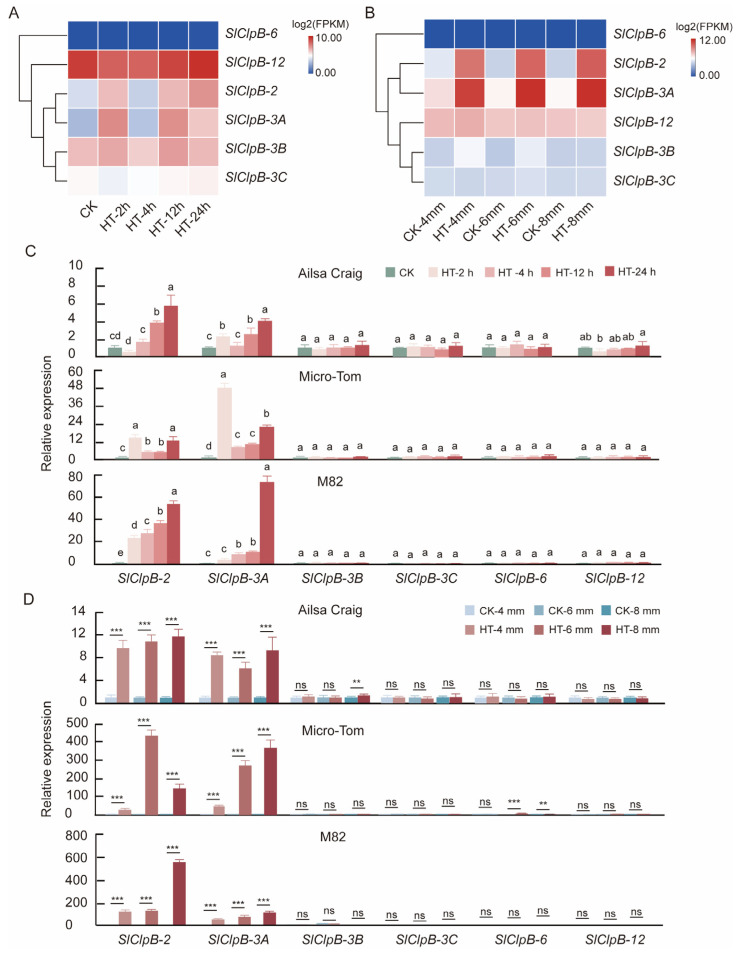
Expression patterns of *SlClpB* genes in tomato under heat stress. (**A**) Expression of *SlClpB* genes in M82 tomato seedlings exposed to 42 °C for 2, 4, 12, and 24 h, as analyzed by RNA-seq. (**B**) Expression in Ailsa Craig flower buds exposed to 25 °C (control, CK) or 37 °C for 0.5 h or 1 h (heat stress, HT), as analyzed by RNA-seq. The samples for HT were mixed samples subjected to 37 °C for 0.5 h and 1 h. (**C**) Expression levels in tomato leaves of Ailsa Craig, Micro-Tom, and M82 exposed to 42 °C for 2, 4, 12, and 24 h, as determined by qRT-PCR. (**D**) Expression levels in flower buds of Ailsa Craig, Micro-Tom, and M82 exposed to 25 °C (control, CK) or 37 °C for 0.5 h or 1 h (heat stress, HT), as measured by qRT-PCR. Different letters indicate statistically significant differences among groups (Tukey’s honestly significant difference test, *p*-value < 0.05). Asterisks indicate significant changes compared to CK (control) samples as calculated by a two-tailed Student’s *t*-test (ns, not significant; ** *p*-value < 0.01; *** *p*-value < 0.001).

In this study, we analyzed the expression of *SlClpB* genes using RNA-seq data from the tomato cultivars M82 and Ailsa Craig under a 42 °C heat stress condition [42,43]. M82 seedlings were subjected to heat stress at 42 °C for different durations (0, 2, 4, 12, and 24 h). *SlClpB-2* and *SlClpB-3A* showed an initial upregulation at 2 h, followed by decreased expression at 4 h, and then a subsequent increase in expression at 12 and 24 h. In contrast, the expression level of *SlClpB-12* decreased by 40.6% at 2 h, followed by no change at 4 h; then, the expression level increased by 57.3% at 12 h and 35.8% at 24 h (Figure 7A). In Ailsa Craig flower buds of lengths 4 mm, 6 mm, and 8 mm, *SlClpB-2* and *SlClpB-3A* also exhibited significant upregulation during heat treatment at 37 °C, while other genes did not show significant expression changes (Figure 7B). These findings imply that the ClpB family plays a key role in tomato responses to high temperatures.

To further validate these findings, we conducted real-time quantitative fluorescent PCR (qRT-PCR) analysis. Under heat stress conditions of 42 °C, we observed a general trend of an increased expression of *SlClpB-2* and *SlClpB-3A* in the leaves of Ailsa Craig, Micro-Tom, and M82 at 2, 4, 12, and 24 h (Figure 7C). In flower buds of Ailsa Craig, Micro-Tom, and M82 at various developmental stages, the expression of *SlClpB-2* and *SlClpB-3A* was significantly elevated under heat stress (Figure 7D). These qRT-PCR results of three tomato genotypes corroborate the RNA-seq data, reinforcing the expression patterns of *SlClpB-2* and *SlClpB-3A* induced under heat stress.

## 3. Discussion

ClpB proteins are crucial for plant growth, development, and adaptation to environmental stresses [13,30]. In this study, we identified six tomato *SlClpB* genes through BLASTp and phylogenetic analysis. These *SlClpB* genes were classified into three distinct clades based on their gene structures (Figure 1). This classification aligns with previous findings in plants, emphasizing the conserved evolutionary patterns of the ClpB family across various plant species [44,45].

However, a homologous gene of the *ClpB* gene specifically localized to the mitochondria was not identified in this study. Initially, we identified a mitochondrial-targeted *ClpB*-like gene (Solyc06g011400), but further investigation revealed that this gene lacks some key structural features typically associated with ClpB proteins [44], particularly the Clp_N domain, a defining characteristic of ClpB proteins [15,16]. The absence of these characteristics could be attributed to several factors, such as limited research, genetic diversity, incomplete genome annotation, species-specific variation, or technical challenges. Additional research is required to confirm the existence and clarify the potential functions of this gene in tomatoes.

Signal peptides are closely related to protein transport [46,47]. However, we identified six *SlClpB* genes in the tomato genome that lack signaling and transit peptides but are predicted to be localized in the chloroplast. This raises an intriguing question about the underlying mechanism by which these proteins enter the chloroplast without conventional import signals. Many cytoplasmic proteins that lack signal peptides are transmitted via unconventional protein secretion (UPS) [48,49,50]. We speculate that the entry may be mediated by UPS, but the specific mechanisms of how UPS involves different routes require further study.

To investigate the evolutionary relationships within the ClpB family across different species, we conducted a gene evolutionary analysis. Two gene pairs identified in the tomato genome appear to have originated from an ancient whole-genome duplication event [51], which likely contributed to the diversification of the *ClpB* gene family (Figure 2A). The Ka/Ks ratio analysis indicates that these genes have been subjected to strong purifying selection, ensuring their functional integrity throughout evolution (Figure 2B). Interspecies collinearity analysis further confirmed the conservation of 13 gene pairs across species, with *SlClpB-6* uniquely present in tomatoes but absent in potatoes and peppers.

Our analysis revealed three distinct domain clusters across all SlClpB proteins: (1) two ATPase associated with diverse function domains (AAA); (2) two Clp amino-terminal domains (Clp_N); and (3) one C-terminal D2-small domain (Clp_D2-Small) (Figure 3). Notably, motifs crucial for ClpB activities are KYRG (pore 1), GYVG (pore 2), and the SSD motif GARPHxRxHx [6,17]. These residues and motifs are important for ClpB functionality, as they affect ATPase activity and substrate binding across diverse plant species. The 3D structure prediction results revealed the protein structure conservation of the SlClpB gene family (Figure 4). These findings imply that the *ClpB* gene family has evolved through both functional conservation and species-specific adaption. Additionally, structural and motif variations among the six *SlClpB* genes (Figure 5) point to potential functional divergence within the family.

Plant hormones are intrinsic signaling molecules that are of paramount importance in regulating plant development, growth, and defense mechanisms [52,53,54,55,56]. Recent studies have found that the exogenous application of plant hormones like abscisic acid, brassinosteroids, gibberellins, auxins, cytokinins, jasmonic acid, and ethylene can significantly mitigate plant heat damage and enhance heat tolerance [57]. Although the SlClpB family is primarily known for its role in preventing protein aggregation and refolding heat-denatured proteins under elevated temperatures, which helps preserve protein functionality and increases heat resistance [58], limited research has been undertaken on the effects of hormones on ClpB proteins. The analysis of the cis-regulatory elements of *SlClpB* genes identified several light-responsive and hormone-responsive elements, with the latter showing the highest diversity (Figure 5). This suggests that *SlClpB* genes may be responsive to various hormone signals in tomatoes, potentially playing a role in the plant’s adaption to increased temperatures.

*SlClpB* genes play a crucial role in the growth and development of plants. Previous studies have identified cytoplasmic ClpB proteins from different plant species and have partially investigated their functions in enhancing heat tolerance [59,60]. In Arabidopsis, *AtClpB1* is implicated in flower development; its knockout results in delayed flowering, while overexpression accelerates this process [61]. Similarly, in rice, *OsHSP101* functions as a key regulator in seed development, collaborating with HSP70cp-2 to control starch biosynthesis and endosperm development [62]. These findings align with our research, reinforcing the role of SlClpB genes in growth and developmental processes. Except for *SlClpB-6*, all *SlClpB* genes exhibit significant expression across multiple tissues, particularly in flower buds and fruits (Figure 5). This dynamic expression pattern suggests that *SlClpB* genes are intricately regulated throughout tomato development, with different family members likely playing distinct roles in various tissues and stages of fruit maturation. These findings emphasize the specialized functions and complex regulatory mechanisms within the SlClpB family.

*AtClpB1* plays a pivotal role in Arabidopsis heat stress [63]. The *ClpB* genes in tomato exhibit significant similarities to their homologs in other plant species, particularly in their roles in stress response and protein homeostasis. Arabidopsis AtClpB1 has been shown to be essential for HSP folding and molecular chaperone activity [64]. Similarly, an increase in the expression of all *TaClpB* members was observed under heat stress, except for *TaCLPB-4B1* in wheat [44]. OsHSP101 co-accumulates with OsHsp16.9A at specific stages of rice seed development and forms a stable complex at high temperatures, playing an important role in improving the heat tolerance of rice [32]. The expression patterns of *ClpB* genes in tomato under heat stress are consistent with those observed in other plant species. The rapid upregulation of *ClpB* genes in response to heat stress has been noted in both *A. thaliana* and rice [32,65]. This coordinated expression likely reflects a conserved mechanism for stress adaptation across plant species.

Mutants lacking *AtClpB1* show decreased heat stress tolerance in comparison to wild-type plants [66]. The introduction of *AtClpB1* cDNA into rice, cotton (*Gossypium hirsutum*), and tobacco (*Nicotiana tabacum*) significantly enhances heat tolerance and improves pollen heat resistance [67]. Similarly, the overexpression of *OsHSP101* in rice boosts heat tolerance under temperatures ranging from 45 °C to 50 °C [65]. In addition to heat tolerance [66], *AtHSP101* also contributes to the overall adaptability of plants [65]. Moreover, HSP101 is present in both Arabidopsis and tomato, and has been indicated to be involved in the chloroplast development and heat resistance of this organelle [29,65]. In tomatoes, *SlClpB-2* and *SlClpB-3A* show significant upregulation in leaves and flower buds under heat stress, highlighting their crucial roles in these tissues during heat stress (Figure 6). These findings suggest that different *SlClpB* genes function distinctively across various tomato tissues when exposed to high-temperature conditions. This variation in expression may reflect the intricate regulatory networks and adaptive mechanisms of *SlClpB* family members during various developmental stages and in different tissues.

## 4. Materials and Methods

### 4.1. Tomato Plant Cultivation and Heat Stress Treatment

The tomato cultivars Ailsa Craig, Micro-Tom, and M82 were used in this study. Seedlings were seeded in 15 cm pots filled with a mixture of peat and vermiculite (2:1, V:V). They were watered daily to maintain optimal moisture levels in the substrate. Seedlings were cultivated in a plant incubator at China Agricultural University, Beijing, under controlled conditions of 25 °C/18 °C (day/night) with a 16 h light/8 h dark photoperiod, providing approximately 400–500 µmol·m^−2^·s^−1^ of light intensity. Seedlings subjected to heat stress were approximately 5 weeks old at the five-leaf stage, while those treated with two flower buds were approximately 7 weeks old.

For heat stress treatment, the five-leaf-stage seedlings were exposed to 42 °C, and leaf samples were at collected at 0 h (control, CK), 2 h, 4 h, 12 h, and 24 h. Additionally, tomato seedlings with two buds were subjected to 37 °C treatment. Bud samples (4 mm, 5 mm, and 6 mm in length) were collected at two time points: 0.5 h or 1 h at 25 °C (control, CK) or 37 °C (heat stress, HT).

### 4.2. Identification and Characterization of SlClpB Genes

The Arabidopsis *AtClpBs* sequences (AT1G7430.1, AT2G25141.1, AT5G15450.1) were used as reference and query sequences to identify homologous sequences in the tomato genome. Homolog searches were conducted using databases from the NCBI (http://www.ncbi.nlm.nih.gov/, accessed 14 May 2024), the Sol Genomics Network (https://solgenomics.net/, accessed 14 May 2024), and Spud DB (http://spuddb.uga.edu/, accessed 14 May 2024). The identification criteria used were those established by Dhaliwal [68], referencing the Tomato Genome Version 4.0. The confirmed SlClpB family members include *SlClpB-2* (Solyc02g088610.4.1), *SlClpB-3A* (Solyc03g115230.3.1), *SlClpB-3B* (Solyc03g117950.3.1), *SlClpB-3C* (Solyc03g118340.3.1), *SlClpB-6* (Solyc06g082560.3.1), and *SlClpB-12* (Solyc12g042060.3.1). The ancestral relationship between these genes and their homologs from other species was confirmed using Ensemble Plants (http://plants.ensembl.org/, accessed on 18 May 2024). Protein sequence molecular weights and isoelectric points (pI) for the six *SlClpB* gene proteins were determined using the proteomic tools available on UniProt (www.uniprot.org, accessed on 18 May 2024). Signal peptide prediction was carried out via Novopro (https://www.novopro.cn/tools/signalp, accessed on 10 August 2024), and subcellular localization predictions were obtained from UniProt (https://www.uniprot.org/, accessed on 10 August 2024).

### 4.3. Motif Analysis of SlClpB Genes

TBtools was used to analyze the conserved motifs of SlClpB proteins. The Batch CD-search tool (https://www.ncbi.nlm.nih.gov/Structure/bwrpsb/bwrpsb.cgi, accessed on 18 May 2024) was employed to predict the domain structures, generating a file named Hitdata. TBtools, designed for visualizing GFF3 files, was used to analyze the genetic structures of the SlClpB genes [69]. Additionally, TBtools extracted 2000 bp promoter sequences from the complete tomato genome. Promoter cis elements in the tomato were predicted using PlantCARE [70] (http://bioinformatics.psb.ugent.be/webtools/plantcare/html/, accessed on 18 May 2024), and visualized through TBtools (https://github.com/CJ-Chen/TBtools/releases, accessed on 18 May 2024).

### 4.4. Phylogenetic Tree Construction of SlClpB Genes

Putative SlClpB gene sequences from various species, including pepper, potato, Arabidopsis, and tomato, were obtained for phylogenetic analysis. The full amino acid sequences of these SlClpB proteins were aligned using the Clustal X 2.1 (https://www.ebi.ac.uk/Tools/msa/clustalo/ accessed on 10 August 2024) [71]. After generating the initial alignment, manual inspection and trimming were performed to remove poorly aligned regions, gaps, and ambiguously aligned sequences that could potentially distort the phylogenetic analysis. The phylogenetic analysis was performed using the maximum likelihood method, RAxML v.8.2.12 (http://epa.h-its.org/raxml/submit_single_gene, accessed on 10 August 2024) on the CIPRES scientific gateway, and the GTR evolutionary model [72]. Bootstrap values from 1000 replicates are shown at the nodes, indicating the reliability of the branching pattern. The selected model was the Poisson model, and the final tree was edited using Adobe Illustrator 2019.

### 4.5. Genome Localization and Synteny Analysis

SWISS-MODEL was used to predict the 3D structure of the SlClpB proteins with I-TASSER (http://zhang.bioinformatics.ku.edu/I-TASSER accessed on 18 May 2024). Synteny analysis and visualization of the *SlClpB* genes were also performed using TBtools [73].

### 4.6. Gene Expression Profile Using RNA-seq Data

RNA-seq data were downloaded from the NCBI Sequence Read Archive (SRA) for the tomato cultivars M82 (PRJNA635375) and Ailsa Craig (PRJNA603594), as well as from the Tom Express database for Micro-Tom (PRJNA307656). Expression data were analyzed using TBtools software [73].

To investigate the expression patterns of *SlClpB* genes in tomatoes under high temperatures, raw RNA sequencing reads were processed [74]. Low-quality reads were filtered using the fastp tool (https://sourceforge.net/projects/project-123ngs/ accessed on 18 May 2024) [75], and high-quality reads were aligned to the tomato reference genome (SL4.0) using Hisat2 [76]. The resulting RNA-seq alignments were subsequently assembled into potential transcripts using StringTie (https://ccb.jhu.edu/software/stringtie/ accessed on 18 May 2024) [77]. The transcript expression levels were normalized to FPKM, followed by Z-score normalization to visualize expression profiles and mitigate potential outliers.

### 4.7. RNA Extraction and qRT-PCR Analyses

RNA was extracted from the leaf and bud samples using an RNA extraction kit (TianGen, Beijing, China). An amount of 1 μg of RNA was subsequently converted into complementary DNA (cDNA) using the HiScript III 1st Strand cDNA Synthesis Kit (Vazyme, Nanjing, China). Real-time fluorescence quantitative PCR (qRT-PCR) was performed by Applied Biosystems 5700 (Thermo Fisher Scientific, Waltham, MA, USA) using TB Green Advantage qPCR premix (TaKaRa, Shiga, Japan). The PCR reaction conditions were as follows: 95 °C for 3 min; denatured at 95 °C for 15 s; annealed at 58 °C for 15 s; extended at 72 °C for 30 s; 40 cycles. The tomato Actin2 gene was the internal reference. The experiment was conducted with three biological replicates per *SlClpB* gene. Data were analyzed using the 2^−ΔΔCt^ method [78]. The primers used for qRT-PCR are provided in Table S3.

## 5. Conclusions

This study identified six *SlClpB* genes in tomatoes and classified them into three subfamilies based on their structural similarity to those in Arabidopsis, highlighting the conserved evolution of the ClpB family across plant species. Tissue-specific expression analyses revealed high *SlClpB* expression levels during fruit and flower development. Notably, significant differences in the expression of *SlClpB-2*, *SlClpB-3A*, and *SlClpB-3B* were observed in seedlings under heat stress, with *SlClpB-2* and *SlClpB-3A* demonstrating higher expression in leaves and flower buds. These findings establish a basis for further investigation into the role of the ClpB family in tomato heat stress tolerance. In the future, it is necessary to verify the function of *SlClpB* genes in heat stress response through gene knockout and overexpression experiments, and to study the interaction network and transcriptional regulation mechanism of SlClpB proteins in heat stress response to reveal their specific action pathways. This will provide theoretical basis and practical guidance for improving the heat resistance of crops.

## Figures and Tables

**Table 1:** The physical and chemical properties of SlClpB family members.

Gene Name	Gene ID	Gene Position		CDS (bp)	AA (aa)	MW (kDa)	pI	Signal Peptide	Prediction of Subcellular Localization
		Start	End						
*SlClpB-2/LeHSP100*	Solyc02g088610	48691924	48699037	3138	980	110.377	6.41	NO	Chloroplast
*SlClpB-3A*	Solyc03g115230	59435365	59439567	3064	911	101.116	6.03	NO	Cytoplasm
*SlClpB-3B*	Solyc03g117950	61374547	61381305	3119	964	105.713	6.88	NO	Cytoplasm
*SlClpB-3C*	Solyc03g118340	67245569	61699244	3146	926	102.61	4.82	NO	Cytoplasm
*SlClpB-6/HSP101*	Solyc06g082560	45913978	45916542	2565	854	95.071	6.31	NO	Cytoplasm
*SlClpB-12*	Solyc12g042060	40789176	56728789	2772	923	102.21	5.99	NO	Cytoplasm

## Data Availability

Data are contained within the article and the Supplementary Materials.

## Supplementary Materials

The following supporting information can be downloaded at: https://www.mdpi.com/article/10.3390/ijms252212325/s1.
